# Novel aptamer-based strategies for addressing aggressive and heterogeneous triple-negative breast cancer

**DOI:** 10.3389/fonc.2026.1943528

**Published:** 2026-10-21

**Authors:** Natassia Silva de Araujo, Aline dos Santos Moreira, Rayane da Silva Abreu, Julia Badaró Mendonça, Tayanne Felippe Sassaro, Wim Degrave, Tatiana Martins Tilli, Mariana Caldas Waghabi

**Affiliations:** 1 Laboratory of Applied Genomics and Bioinnovations, Oswaldo Cruz Institute, Fiocruz, Rio de Janeiro, RJ, Brazil; 2 Laboratory of Clinical and Experimental Pathophysiology, Oswaldo Cruz Institute, Fiocruz, Rio de Janeiro, RJ, Brazil; 3 Translational Oncology Platform, Center for Technological Development in Health, Fiocruz, Rio de Janeiro, RJ, Brazil

**Keywords:** AptaBreast panel, aptamers, breast cancer, therapy, triple-negative

## Abstract

**Introduction:**

Triple-negative breast cancer (TNBC) is a highly aggressive and heterogeneous subtype of breast cancer associated with poor prognosis, limited therapeutic options, and high recurrence rates.

**Methods:**

In this study, we evaluated the therapeutic potential of an AptaBreast panel (AB-panel) previously identified for its ability to recognize TNBC cells specifically. Functional assays were performed in various TNBC cell lines representing distinct intrinsic subtypes, as well as in non-tumoral cells (MCF10A).

**Results:**

Functional assays revealed that the aptamers effectively reduced tumor cell viability, proliferation, migration and colony formation in various TNBC cell lines. Importantly, the combined treatment with all five aptamers resulted in an enhanced overall antitumor effect. Notably, the aptamers demonstrated selective action on tumor cells without significant cytotoxicity to non-tumoral cells (MCF10A).

**Discussion:**

The observed effects support the potential of these aptamers as therapeutic candidates for TNBC and warrant further investigation into their molecular targets and mechanisms of action. Collectively, our findings highlight their potential across different TNBC cellular models, while further studies are needed to assess their therapeutic efficacy, safety, and applicability in more complex models. These results provide a basis for future preclinical studies aimed at further evaluating their potential in TNBC.

## 1 Introduction

Breast cancer is the most common type of cancer among females and the leading cause of mortality in women (1). The disease can be stratified into subtypes based on the expression of receptors found on the membrane of breast tumor cells (2). Among these subtypes, triple-negative breast cancer (TNBC) stands out as the most aggressive molecular subtype, commonly associated with metastases. It is more prevalent among young women and accounts for approximately 15% to 20% of all breast cancer diagnoses (3). The hallmark of TNBC is the lack of expression of the progesterone receptor (PR), estrogen receptor (ER), and human epidermal growth factor receptor 2 (HER2) (4). Moreover, TNBC is characterized by high heterogeneity and intrinsic subdivisions based on the molecular profile of tumor cells, which can be categorized into basal-like (BL1 and BL2), mesenchymal (M), immunomodulatory (IM), mesenchymal stem-like (MSL), and luminal androgen receptor (LAR) subtypes (5).

Mortality from breast cancer is often linked to the development of metastases in the bones, lungs, and brain (6). TNBC is commonly treated with cytotoxic chemotherapy. However, traditional cancer treatments, such as chemotherapy, frequently prove insufficient for advanced and aggressive tumors. This inadequacy is primarily due to their limited specificity, leading to high recurrence rates and significant toxicity (7). In recent years, more targeted cancer therapies have become available, such as monoclonal antibodies used in immunotherapies. Nonetheless, some patients have tumors that are unresponsive to this approach, and such therapies fail (8). In the quest for innovative strategies in targeted therapy, nanotechnology tools such as aptamers have emerged as promising alternatives.

Aptamers are synthetic oligonucleotides composed of either DNA or RNA. They belong to a class of ligands formed by single-stranded sequences that fold into specific three-dimensional structures. These unique structures allow aptamers to bind selectively and with high affinity to a wide range of biologically relevant molecules (9). The interaction between an aptamer and its target molecule occurs primarily through hydrogen bonding, which facilitates electrostatic interactions. Aptamers can vary in size but typically include a central random region, capable of up to 10^^16^ possible sequence combinations, flanked by two constant regions that enable their synthesis through PCR amplification (10, 11).

Due to their functional similarities, aptamers are often compared to antibodies and are sometimes referred to as “synthetic antibodies”. However, aptamers offer several advantages over antibodies, such as ease of development in molecular laboratories, the absence of animal use in their production - removing the risk of cross-contamination - and reduced production costs and complexity (12). Furthermore, aptamers are smaller than antibodies, which enhances tissue penetration (13).

The aptamer can directly target cancerous cells by acting as an antagonist to key receptors involved in tumor cell development and progression or by being used in combination with pharmacological agents. Their application depends on various factors, including the selection method, the target molecule, structural properties, and the potential for conjugation with other molecules (14).

In a previous study conducted by our group, using the cell-SELEX methodology, we selected five aptamers highly specific for breast cancer cells (15). In the present study, we evaluated the ability of this panel of aptamers, referred to as AptaBreast panel (AB-panel), to generate antitumor effects in triple-negative breast cancer cell lines by inducing cell death, inhibiting proliferation, and suppressing tumor cell migration.

## 2 Materials and methods

### 2.1 Cell lines and culture conditions

The triple-negative breast cancer cell lines MDA-MB-231, MDA-MB-468, HCC-70, and HCC-1937 were maintained in RPMI 1640 medium supplemented with 10% fetal bovine serum and 100 U/mL penicillin/streptomycin (Sigma, P4333). The non-tumoral cell line MCF10A was cultured in MEGM™ Mammary Epithelial Cell Growth Medium (Lonza/Clonetics Corporation, CC-3150 - MEGM BulletKit) supplemented with BPE, rhEGF, hydrocortisone, insulin, 100 U/mL penicillin/streptomycin (Sigma, P4333), and 10 ng/mL cholera toxin (Sigma, C8052). All cell lines were authenticated and routinely tested for mycoplasma contamination before use (data not shown).

### 2.2 Trypan blue exclusion method

To evaluate the cytotoxicity of the aptamers, MDA-MB-231 cells were plated in 48-well plates at a density of 2.5 × 10^4^ cells per well. After 24 hours, the cells were treated with 400 nM of individual aptamers or a combination of all five aptamers (400 nM each). This experiment was conducted kinetically, with observations at 24, 48 and 72 hours post-treatment. At each time point, the cells were dissociated, diluted in 0.4% trypan blue, and both viable and dead cells were quantified using the Countess II automated cell counter (ThermoFisher). Triton X-100 (0.01%), a membrane-disrupting detergent, was used as a positive control to disrupt cell membrane integrity, allowing Trypan blue uptake and resulting in the identification of these cells as non-viable. Three independent assays were performed, each in triplicate.

### 2.3 Cell viability using PrestoBlue reagent™

To assess the viability of tumor and control cells treated with aptamers, a resazurin-based redox assay was performed using the PrestoBlue reagent (ThermoFisher). For this assay, 4 × 10^4^ cells from triple-negative cell lines (MDA-MB-231, MDA-MB-468, HCC-70, and HCC-1937) and a non-tumoral cell line (MCF10A) were plated in duplicate in 96-well plates. After 24 hours of plating, cells were treated with 400 nM of individual aptamers or a combination of all aptamers (400 nM each). RPMI medium and a random-sequence aptamer library were used as negative control. Cell viability was evaluated at 24, 48 and 72 hours post-treatment. At each time point, 10 μL of PrestoBlue reagent was added to each well, and the plates were incubated for 1 hour at 37 °C in a CO_2_ atmosphere. Fluorescence readings were performed using the Varioskan Lux equipment (ThermoFisher) at excitation and emission wavelengths of 560 nm and 590 nm, respectively. Triton X-100 (0.01%), a membrane-disrupting detergent, was used as a positive control to disrupt cell membrane integrity and induce loss of cell viability. Three independent assays were performed in triplicate for the MDA-MB-231, MDA-MB-468, and MCF10A cell lines, and in duplicate for the HCC-70 and HCC-1937 cell lines.

### 2.4 Cell proliferation assay - CyQUANT™

To assess the ability of aptamers to inhibit cell proliferation, the CyQUANT™ Cell Proliferation Assay Kit (Molecular Probes) was used. This assay utilizes a dye with high affinity for nucleic acids to measure the DNA content in the sample. For the assay, 100 μL of the CyQUANT working solution was added to each well, and the plates were incubated at 37 °C in a CO_2_ atmosphere for 30 minutes. Fluorescence readings were performed using the Varioskan Lux equipment (ThermoFisher), available at the Bioassays Platform of IOC/Fiocruz. Triton X-100 (0.01%), a membrane-disrupting detergent, was used as a positive control to disrupt cell membrane integrity, resulting in loss of cell viability and proliferative capacity. Three independent assays were performed in triplicate for the MDA-MB-231, MDA-MB-468, and MCF10A cell lines, and in duplicate for the HCC-70 and HCC-1937 cell lines.

### 2.5 Colony formation assay

To assess the ability of aptamers to inhibit colony formation in the MDA-MB-231 tumor cell line, cells were initially plated at a density of 2.5 × 10^4^ cells per well in 48-well plates treated with 400 nM of individual aptamers or a combination of all aptamers (400 nM each) for 24 hours. After treatment, cell viability was assessed, and the total number of cells replated was proportionally adjusted according to the viability determined for each treatment condition, aiming to obtain an estimated 250 viable cells per well. Cells were then replated in 6-well plates. The culture was maintained for 9 days, with replacement of the medium every 48 hours. At the end of the incubation period, colonies were fixed with 4% paraformaldehyde for 5 minutes at room temperature and stained with 0.5% crystal violet for 20 minutes. Colony numbers were manually counted. This normalization was performed to minimize differences in colony formation attributable to the initial cytotoxic effect of the treatment and to enable comparison of the clonogenic potential of the surviving cell population.

### 2.6 Migration assay

To evaluate the migration of MDA-MB-231 cells, Transwell insert chambers (Corning, Corning, NY) were used. After 24 hours of incubation with aptamers, treated and control cells were dissociated following 24 hours of serum deprivation. A total of 5 × 10^4^ cells were plated in the upper chamber in serum-free RPMI medium, while RPMI medium supplemented with 10% FBS was added to the lower chamber as a chemoattractant. After 24 hours of incubation, the medium was removed from both the upper and lower chambers. Cells on the insert were fixed with 4% formaldehyde for 10 minutes and stained with 0.4% crystal violet for 15 minutes. The inserts were washed twice with PBS, and non-migrated cells on the upper side of the Transwell membrane were carefully removed using a cotton swab. At least five images were captured for each sample using a Motic microscope, and the number of migrated cells was quantified using ImageJ software.

### 2.7 Statistical analysis

Statistical and graphical analyses of the data were performed using GraphPad Prism 5 software. All experiments were independently performed in biological triplicate (n = 3). For statistical analysis, one-way analysis of variance (ANOVA) followed by Tukey’s *post-hoc* test was applied.

## 3 Results

### 3.1 Evaluating the cytotoxic effects of the AptaBreast panel on tumor cell viability

After investigating the ability of aptamers to recognize TNBC cell lines and breast tissues, their functional role in inducing cell death was evaluated. Negative control wells received only RPMI medium or the aptamer library, while Triton X-100 (0.01%) served as a positive control for cell viability reduction. Viable and non-viable cells were analyzed at 24, 48 and 72 hours following incubation with the aptamers. Cells were dissociated, incubated with 0.4% trypan blue, and the percentage of viable and non-viable cells was quantified using the Countess II automated cell counter.

The results showed that aptamers induced cell death in 40% to 57% of treated cells within 24 hours. Treatment with the combination of all five aptamers resulted in ~70% cell death, suggesting an additive effect. After 48 hours of incubation, a reduction in cell viability of 36% with AptaB2 and 64% with AptaB4 was observed. Additionally, the combination of all aptamers induced 76% cell death at this time point. At 72 hours, approximately 60% cell death was observed with individual aptamers, while the combination of all aptamers led to 84% cell death (**Figures 1A, B**), as compared to 12% with RPMI and 10% with the random aptamer library.

**Figure 1:**
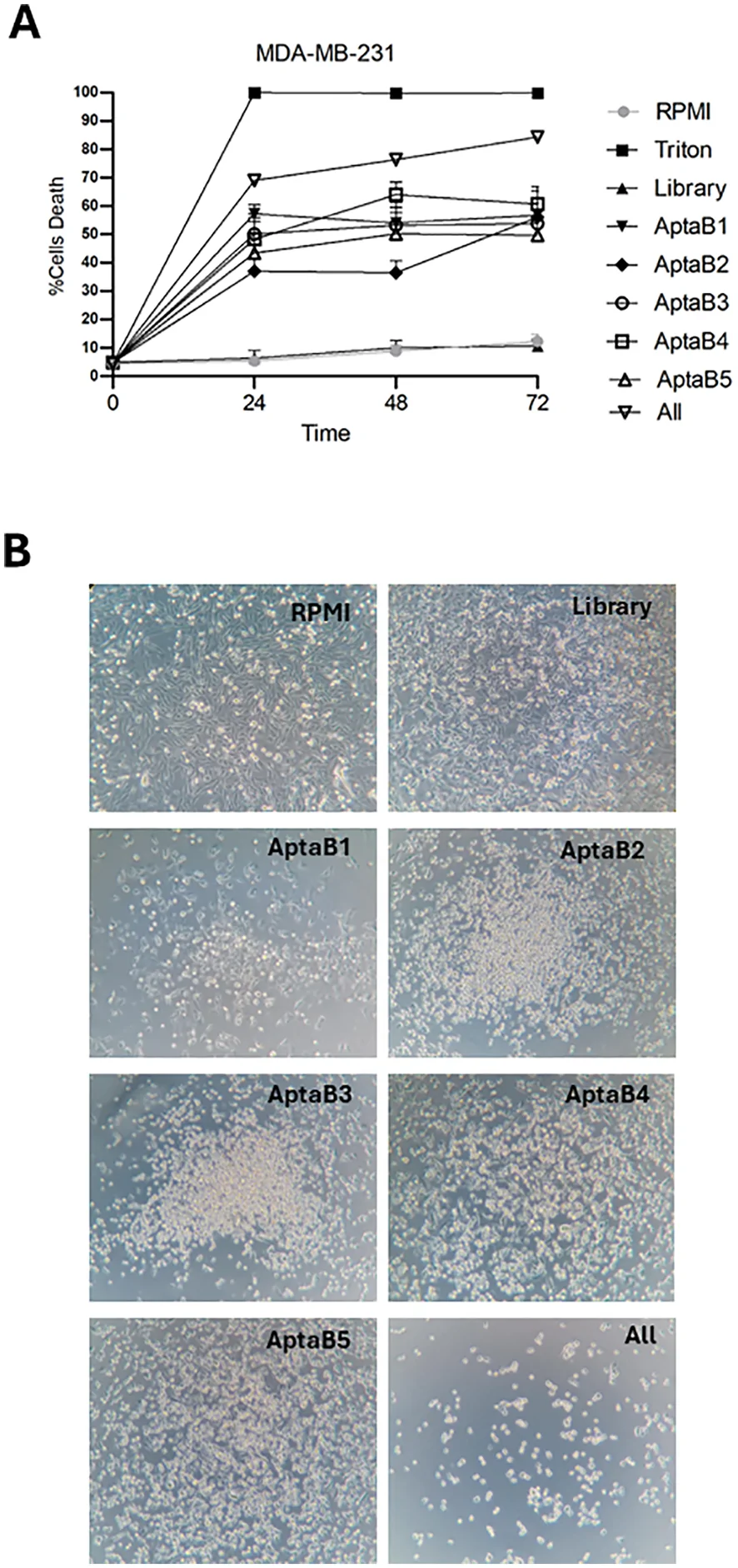
Effect of AptaBreast panel **(AB**-panel) treatment on the viability of MDA-MB-231 cells using the trypan blue exclusion method. **(A)** Graph showing the percentage of dead cells at 24, 48 and 72 hours after treatment with individual aptamers (AptaB1–AptaB5) or the combination of all five aptamers. **(B)** Representative images of treated and control cells after 72 hours, highlighting the morphological changes induced by aptamer treatment.

### 3.2 Assessment of AptaBreast panel-induced effects on cell viability across TNBC and non-tumoral cell lines

Following the observation that aptamer treatment decreases cell viability of targeted tumor cells, a second functional assay was conducted using the PrestoBlue™ Cell Viability Reagent. This assay measures the ability of live cells to metabolize resazurin into the fluorescent compound resorufin. In addition to the MDA-MB-231 cell line, the effects of aptamers on other triple-negative breast cancer cell lines and the non-tumoral MCF10A cell line were evaluated. Cells were plated in 96-well plates and were treated after 24 hours with 400 nM of each aptamer individually or a combination of all five. Negative control wells received only RPMI medium or the aptamer library, while Triton X-100 (0.01%) served as a positive control for cell viability reduction. Analyses were performed at 24, 48 and 72 hours post-treatment.

At 24 hours, MDA-MB-231 cells treated with individual aptamers exhibited a 27% to 37% reduction in viability, while the combination of all five aptamers resulted in a 43% loss of viability. This reduction remained at 48 and 72 hours for both individual and combined treatments (**Figure 2**). In MDA-MB-468 cells, individual aptamers reduced viability by 25% to 32% after 24 hours, while the combination of all five aptamers decreased viability by 58%. After 48 hours, individual aptamers reduced viability by 8% to 27%, while the combined treatment led to a 51% reduction. At 72 hours, these trends persisted, except for the combined treatment, which resulted in a 41% reduction in viability (**Figure 2**).

**Figure 2:**
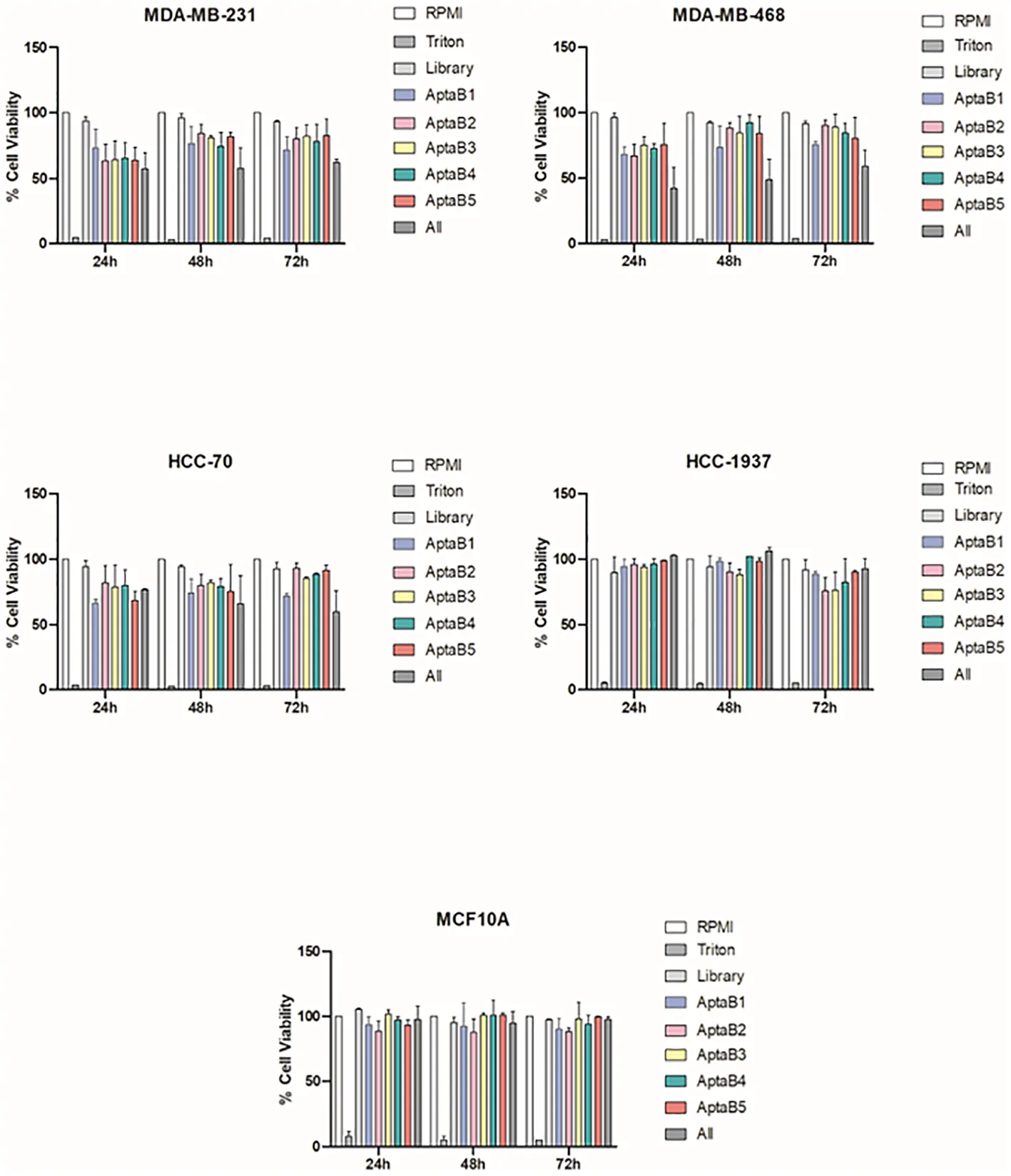
Effect of AptaBreast panel (AB-panel) treatment on triple-negative breast cancer cell lines and the non-tumoral cell line MCF10A, assessed using the PrestoBlue assay. Triple-negative breast cancer cell lines (MDA-MB-231, MDA-MB-468, HCC-70, and HCC-1937) and the non-tumoral MCF10A cell line were treated with individual aptamers (AptaB1, AptaB2, AptaB3, AptaB4, and AptaB5) or the combination of all five aptamers at a concentration of 400 nM. Cell viability was evaluated at 24, 48, and 72 hours post-treatment.

In HCC-70 cells, a 20% to 32% reduction in viability was observed with both individual and combined aptamer treatments after 24 hours. At 48 hours, individual aptamers caused a 25% reduction, while the combined treatment led to a 35% reduction. After 72 hours, AptaB1 reduced viability by 29%, while AptaB2 induced a striking 93% reduction. The combination of all aptamers decreased viability by 46% (**Figure 2**).

For the HCC-1937 cell line, no significant effect was observed after 24 and 48 hours of treatment with individual or combined aptamers. However, at 72 hours, AptaB2 and AptaB3 reduced cell viability by 30% (**Figure 2**). In the non-tumoral MCF10A cell line, a slight reduction in viability (7% to 12%) was observed with AptaB1 and AptaB2. The other aptamers and the combined treatment did not significantly affect the cell viability of the non-tumoral breast cell line (**Figure 2**).

### 3.3 Evaluation of the AptaBreast panel effect on cell proliferation across multiple tumoral and non-tumoral breast cell lines

In addition to evaluating the role of aptamers as inducers of cell death, their effect on cell proliferation was also assessed. For this purpose, the CyQUANT™ Cell Proliferation Assay Kit, which measures DNA content, was utilized. As in the previous assays, cells were treated with 400 nM of each aptamer individually or with a combination of all five aptamers. In the MDA-MB-231 cell line, a reduction in the proliferation index ranging from 11% to 32% was observed after 24 hours of treatment with individual aptamers, while the combination of all five aptamers resulted in a 33% reduction. This effect was sustained after 48 and 72 hours of incubation with either individual or combined treatments (**Figure 3**).

**Figure 3:**
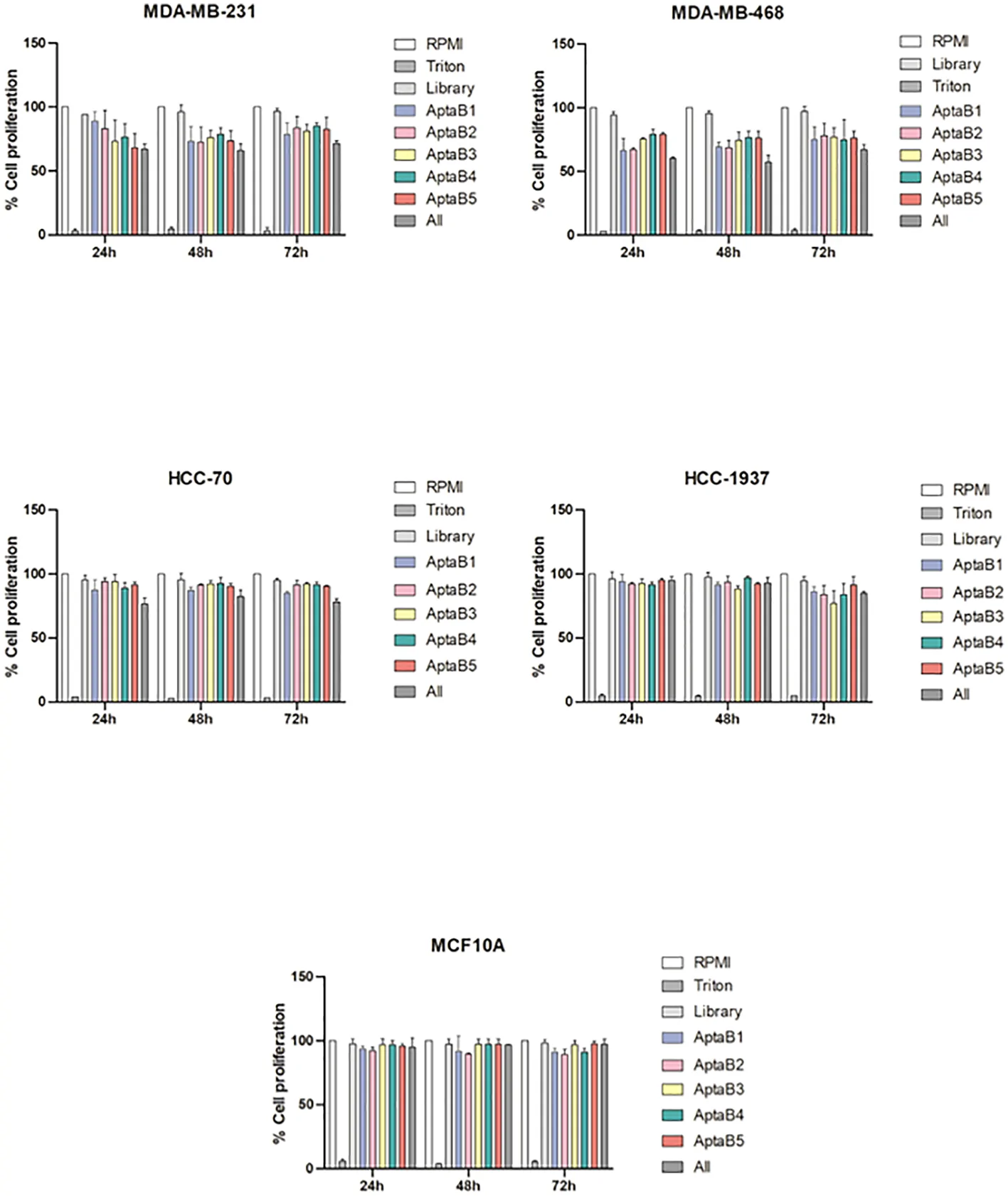
Effect of AptaBreast panel (AB-panel) treatment on triple-negative breast cancer cell lines and the non-tumoral cell line MCF10A, assessed using the CyQuant assay. Triple-negative breast cancer cell lines (MDA-MB-231, MDA-MB-468, HCC-70, and HCC-1937) and the non-tumoral MCF-10A cell line were treated with individual aptamers (AptaB1, AptaB2, AptaB3, AptaB4, and AptaB5) or the combination of all five aptamers at a concentration of 400 nM each. Cell proliferation was evaluated at 24, 48 and 72 hours post-treatment.

In assays using the MDA-MB-468 cell line, a reduction in the proliferation index of 21% to 34% was observed 24 hours after treatment with individual aptamers. The combination of all five aptamers resulted in a 40% reduction in proliferation. This reduction persisted for 48 hours but was lost after 72 hours, when individual treatments led to a decrease of approximately 20%, and the combination of all five aptamers resulted in a 33% reduction (**Figure 3**).

The results obtained with the HCC-70 cell line showed a reduction in the proliferation index of 6% to 13% after 24 hours of individual treatment, while the combination of all five aptamers reduced proliferation by 20%. This effect was maintained after 48 and 72 hours of incubation with either individual aptamers or the combination (**Figure 3**). In assays with the HCC-1937 cell line, a reduction of 9% and 12% in the proliferation index was observed at 24 and 48 hours with both individual and combined treatments. After 72 hours, a 23% reduction was seen in cells treated with AptaB3 (**Figure 3**).

The assays using the non-tumoral MCF10A cell line indicated that at all observed time points, treatment with AptaB1 and AptaB2 resulted in a reduction of approximately 10% in the proliferation index. Other aptamers and the combined treatment did not affect cell proliferation in the non-tumoral control cells (**Figure 3**).

### 3.4 AptaBreast panel as inhibitor of tumor colony formation: a significant reduction in proliferative capacity

After observing a reduction in the proliferation index of tumor cells, a colony formation assay was performed to evaluate the effect of aptamers on the ability of tumor cells to proliferate and form colonies. Following 24 hours of incubation, control and treated cells were counted and re-plated. Colony formation was assessed after 10 days. Treatment with individual aptamers, as well as the combination of all aptamers, significantly reduced colony formation in MDA-MB-231 cells.

The cells treated with AptaB1, AptaB2, and AptaB5 formed less than half the number of colonies compared to the untreated control. Notably, cells treated with AptaB4 or the combination of all aptamers demonstrated the strongest inhibitory effects on colony formation (**Figure 4**), with reductions of up to 86%.

**Figure 4:**
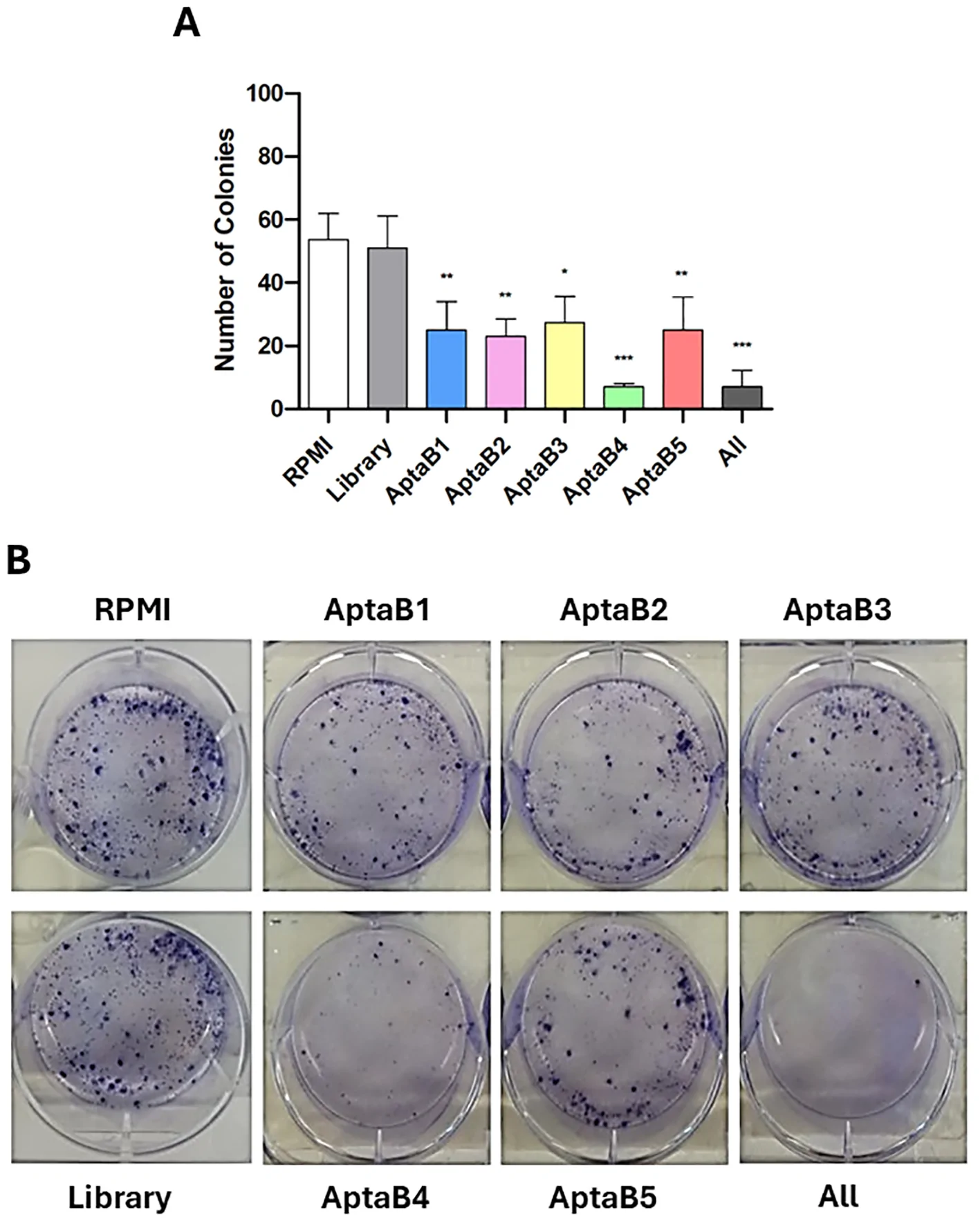
Analysis of colony formation in MDA-MB-231 cells after AptaBreast panel (AB-panel) treatment. **(A)** Quantification of colony numbers following 10 days of treatment with individual aptamers (AptaB1, AptaB2, AptaB3, AptaB4, and AptaB5) or the combination of all five aptamers. **(B)** Representative images of colony growth in untreated and treated MDA-MB-231 cells after 10 days. Statistical significance: *p ≤ 0.05, **p ≤ 0.01, ***p ≤ 0.001.

### 3.5 AptaBreast panel as an effective inhibitor of MDA-MB-231 cell migration

The ability of aptamers to inhibit the migration of MDA-MB-231 cells was assessed as described in the Materials and Methods section. The evaluation revealed that the entire panel of aptamers effectively inhibited cell migration. As shown in the graph in **Figure 5A**, AptaB2 reduced cell migration by 83%, while AptaB1 and AptaB4 achieved reductions of 71% and 64%, respectively. Aptamers AptaB3 and AptaB5 decreased migration by approximately 50%. Treatment with the combination of all aptamers resulted in a 60% reduction in MDA-MB-231 cell migration. Representative images of the migrated cells through the transwell membrane are presented in **Figure 5B**.

**Figure 5:**
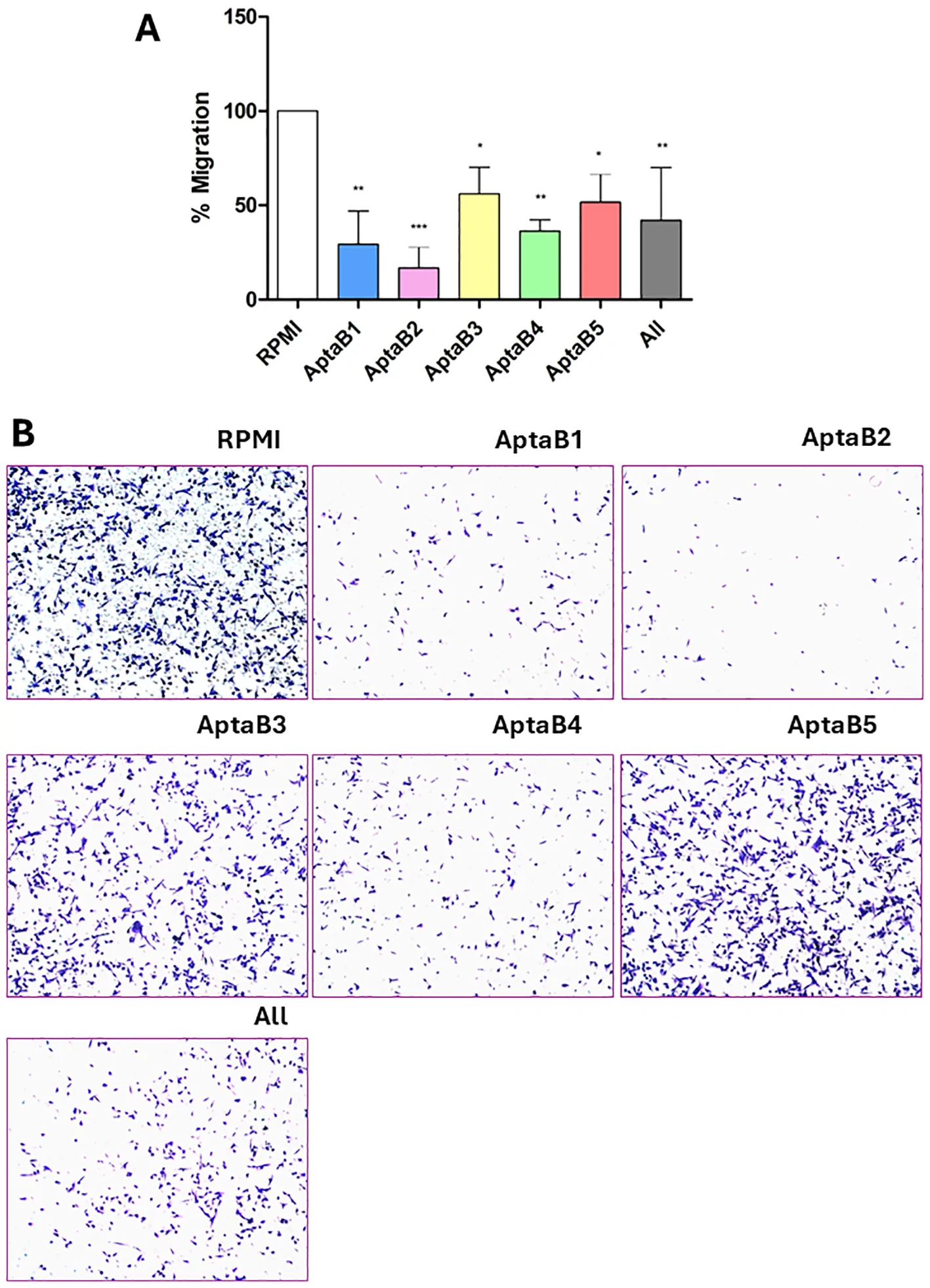
Evaluation of the effect of AptaBreast panel (AB-panel) on the migration of MDA-MB-231 cells. **(A)** Quantification of migrated cells, represented as a graph. **(B)** Representative images showing the migration of untreated MDA-MB-231 cells and those treated with individual aptamers (AptaB1, AptaB2, AptaB3, AptaB4, and AptaB5) or the full aptamer panel. Statistical significance: *p ≤ 0.05, **p ≤ 0.01, ***p ≤ 0.001.

## 4 Discussion

Breast cancer remains a complex and heterogeneous disease, with an average patient survival rate of 18–24 months, which varies depending on its molecular characteristics and pathological staging (16). TNBC is associated with a higher risk of metastasis, an increased likelihood of recurrence, and generally lower survival rates (17, 18). The absence of hormone receptors (ER/PR), as well as the human epidermal growth factor receptor (HER2), excludes the possibility of hormonal therapy or targeted treatment via the HER2 pathway (19). Additionally, the TNBC subtype is often linked to higher drug resistance (20). In a previous study, we described a panel of aptamers selected using the MDA-MB-231 tumor cell line and demonstrated their potential for diagnosing breast cancer cells (15). Here, we present an evaluation of important functional properties of these aptamers and explore their potential as therapeutic agents.

To evaluate the antitumor potential of the AptaBreast panel against TNBC cells, we utilized cell lines representing distinct intrinsic subtypes. The MDA-MB-468 and HCC-1937 cell lines represent the Basal-Like 1 (BL1) subtype, while the HCC-70 cell line corresponds to the Basal-Like 2 (BL2) subtype. Together, BL1 and BL2 account for approximately half of TNBC cases. Additionally, we included the MDA-MB-231 cell line, which represents the mesenchymal (M) subtype, recognized as the most aggressive subtype (21).

The results presented in this study underscore the antitumor potential of aptamers AptaB1-AptaB5 in triple-negative breast cancer cell lines. Viability assays performed with the MDA-MB-231 and MDA-MB-468 cell lines revealed a significant reduction in cell viability within the first 24 hours of treatment. Notably, the combined treatment with all five aptamers maintained this effect up to 72 hours. In assays involving the HCC-70 cell line, AptaB1 exhibited efficacy comparable to that of the combined treatment. While the impact of individual aptamers varied depending on the treatment duration, the most pronounced effect was achieved with the combined treatment, resulting in approximately a 40% reduction in cell viability across the three tested cell lines after 72 hours.

The evaluation of antiproliferative activity revealed up to a 30% reduction in cell proliferation across the three TNBC cell lines analyzed (MDA-MB-231, MDA-MB-468, and HCC-70) following treatment with the aptamer combination, consisting of 400 nM of each aptamer (2 μM total aptamer concentration). The concentration of 400 nM per aptamer was selected based on previously published cellular binding data from our group (15). In that study, the binding of individual aptamers was evaluated across increasing concentrations of 25, 50, 100, 200, and 400 nM, demonstrating a progressive increase in cellular recognition and identifying 400 nM as the optimal concentration among those tested. Based on these findings, 400 nM was selected as the concentration for each aptamer in the combination treatment used in the present study.

The observed reduction in proliferation following combined treatment, compared with the effects previously observed with individual aptamers at 400 nM, may indicate a potential advantage of simultaneously targeting multiple molecular determinants or cellular pathways. However, this interpretation should be made cautiously, as the combination treatment also resulted in a higher total nucleic acid concentration (2 μM) than that used for individual aptamer treatments (400 nM). Therefore, the enhanced antiproliferative effect cannot be unequivocally attributed to a synergistic or multi-target mechanism based solely on these experiments. Nevertheless, the selection of 400 nM per aptamer was grounded in the previously established cellular binding profile, providing a concentration at which efficient cellular recognition had been demonstrated. Further studies using appropriate dose-matched controls and formal combination-effect analyses will be necessary to determine whether the observed response reflects additive, synergistic, or independent effects of the individual aptamers.

Interestingly, no significant treatment effect was observed in the HCC-1937 cell line, although it belongs to the same intrinsic subtype as the MDA-MB-468 cell line. The discrepancy in outcomes between these two cell lines has been previously reported in the literature. For instance, in an assay utilizing tamoxifen to treat TNBC cell lines, apoptosis was induced in the MDA-MB-468 cell line, whereas the HCC-1937 cell line demonstrated insensitivity to the treatment (22). The HCC-1937 cell line, isolated from a primary breast tumor, from a young, premenopausal women (23). In contrast, the MDA-MB-468 cell line was derived from pleural effusion from a 51-year-old postmenopausal woman (24, 25).

In BL1 subtype cells (MDA-MB-468, HCC-1937), deletions in repair genes such as *RB1*, *BRCA* and *TP53* are commonly observed, along with amplifications in oncogenes like *MYC* and *KRAS*, which drive cell replication (26, 27). In the BL2 subtype (HCC-70), activation of growth factor-related pathways and TGF-β signaling is prominent (28, 29). The mesenchymal (M) subtype (MDA-MB-231) is characterized by heightened activity in actin-associated pathways, which enhance cell migration (26, 28). Notably, the M and BL2 subtypes are associated with an increased risk of disease recurrence within three years (30).

Taken together, these findings demonstrate that the AptaBreast panel can exert biological activity across distinct TNBC cellular models, including cell lines representing different molecular subtypes. Nevertheless, the marked molecular heterogeneity of TNBC, which can also occur among cell lines classified within the same intrinsic subtype, poses a significant challenge for the development of broadly effective targeted approaches. Given the high molecular specificity of aptamers, their biological activity may depend on the presence, abundance, accessibility, or molecular context of their cognate targets, potentially resulting in heterogeneous responses across TNBC models. In this regard, the lack of response observed in HCC-1937 cells, despite their classification within the same BL1 subtype as MDA-MB-468 cells, may indicate differences in the expression or availability of the molecular targets recognized by the aptamers across the same intrinsic subtype. Ongoing efforts to identify and validate the molecular targets of the AptaBreast aptamers may provide important insights into the mechanisms underlying this differential response, including whether differences in target expression or accessibility, or alterations in associated molecular pathways, contribute to the observed lack of response. Further studies will also investigate the mechanisms underlying the reduction in cell viability induced by the AptaBreast panel, including the characterization of cell death pathways and effects on cell-cycle progression, as well as further defining the dose–response and IC_50_/EC_50_ values. Collectively, these studies will help elucidate the molecular determinants of aptamer sensitivity and provide a more comprehensive understanding of their mechanisms of action, supporting the rational development of aptamers as precision-targeting agents for heterogeneous TNBC. To evaluate potential cytotoxic effects on non-tumoral cells, we included the MCF10A cell line in our analysis and observed no significant impact on these control cells. Our findings are also consistent with previous studies investigating the biological effects of aptamers in TNBC models. For example, a study investigating the optimized MNK1b-targeting aptamer apMNKQ2 reported a greater reduction in cell viability in MDA-MB-468 cells compared with MDA-MB-231 cells. These findings demonstrate that the same aptamer may exhibit a differential magnitude of effect depending on the intrinsic subtype of TNBC, further emphasizing the importance of considering tumor heterogeneity when evaluating aptamer activity (16). This finding is consistent with the effect of the aptamers on tumor cells. However, beyond evaluating their effects on tumor cell viability and proliferation, it is important to consider their potential impact on cellular processes associated with tumor progression.

This finding aligns with the specificity of the aptamers for tumor cells. According to the literature, more than 40% of TNBC cases progress to metastatic disease, highlighting the aggressive nature of this subtype (31, 32). Metastasis formation occurs when tumor cells from the primary tumor invade the surrounding stroma and bloodstream, subsequently colonizing distant organs such as the brain and lungs (33, 34). To investigate whether aptamers can inhibit the colony-formation ability of tumor cells, we employed the cell line MDA-MB-231 in a colony formation assay. This assay evaluates the *in-vitro* capacity of individual cells to proliferate and establish colonies (35).

Among the TNBC cell lines evaluated in this study, MDA-MB-231 is characterized by a mesenchymal phenotype and has been described as exhibiting greater invasive capacity, a more pronounced stem-like profile, and features associated with increased therapeutic resistance (21, 26, 28). These characteristics make this cell line particularly suitable for investigating functional properties related to tumor aggressiveness, including cell migration and the ability of surviving cells to form colonies following treatment. Therefore, MDA-MB-231 was selected as the initial model for these functional assays. Nevertheless, further studies will be necessary to determine whether these effects are also observed in other TNBC cell lines.

The optimized DNA aptamer apMNKQ2 has also been reported to reduce the migratory and clonogenic capacities of MDA-MB-231 cells (16), supporting the potential of aptamers to interfere with functional properties associated with TNBC aggressiveness. Unlike apMNKQ2, which has a characterized molecular target, the AptaBreast panel comprises five distinct aptamers whose targets remain unknown. Thus, the use of multiple distinct aptamers may increase the likelihood of recognizing different molecular targets on TNBC cells and, consequently, affecting distinct cellular processes. Such a multi-target strategy could potentially reduce the possibility of tumor cell escape associated with alterations in or loss of a single target. However, whether the AptaBreast aptamers recognize different targets and whether this contributes to their biological effects remain to be experimentally determined. This possibility warrants further investigation in light of the differential effects observed among the individual aptamers in the functional assays.

Interestingly, the functional assays of the AptaBreast panel revealed a differential pattern of activity among the individual aptamers. While the viability and proliferation assays showed relatively similar effects among the aptamers, with overall reductions in tumor cell viability and proliferation, more pronounced differences emerged when colony formation and migration were evaluated. In the colony formation assay, all aptamers inhibited colony formation, with AptaB4 showing the most pronounced effect, together with the combination of all aptamers. Similarly, in the transwell migration assay, all aptamers significantly reduced the migratory capacity of MDA-MB-231 cells, although AptaB1 and AptaB2 produced the strongest effects, reducing migration by more than 70%.

The distinct activity profiles observed across these functional assays suggest that the effects of the aptamers on tumor cell behavior may not be solely attributable to their antiproliferative or cytotoxic effects. If the inhibition of migration and colony formation were primarily a consequence of reduced cell viability or proliferation, a more consistent pattern of activity among the aptamers would be expected across these assays. Instead, the stronger effects of AptaB4 on colony formation and of AptaB1 and AptaB2 on cell migration suggest that individual aptamers may interfere with cellular processes specifically associated with clonogenic potential and tumor cell motility. These effects could potentially involve pathways regulating cell adhesion, cytoskeletal organization, epithelial–mesenchymal plasticity, or other mechanisms associated with aggressive tumor phenotypes. Further characterization of the molecular targets and downstream pathways affected by each aptamer will be important to determine whether these differential functional effects are associated with specific molecular mechanisms. Such studies may help establish whether the biological activity of individual aptamers extends beyond direct effects on tumor cell survival and proliferation to modulate cellular properties associated with tumor progression and metastatic potential.

These findings are also consistent with previous observations from our group regarding the ability of the AptaBreast aptamers to recognize tumor tissues with different histological grades. In a previous study, AptaB1, AptaB2, and AptaB4 successfully recognized tumor samples classified as histological grades 2 and 3 (15). Literature evidence suggests that tumors with higher histological grades are generally associated with increased tumor aggressiveness, recurrence, and metastatic potential (36), the recognition of these samples, together with the functional effects observed in the present study, further supports the potential of these aptamers as candidates for investigation in the context of tumor progression and metastasis. However, whether tissue recognition and the functional effects observed *in vitro* are mechanistically related remains to be established.

Despite significant advances in TNBC treatment, this subtype continues to be associated with poor clinical outcomes, and its substantial molecular heterogeneity remains a major challenge for the development of effective therapeutic strategies (37). This challenge is particularly relevant for refractory disease, for which treatment options remain limited and there is no consensus regarding the optimal therapeutic approach in severely debilitated patients (38). In this context, the ability of the AptaBreast panel to induce biological effects *in vitro* across different TNBC intrinsic subtypes is of particular interest, as it may support the development of strategies capable of addressing intertumoral heterogeneity, a defining feature of this disease (39, 40).

Beyond their direct antitumor effects, the molecular recognition properties of aptamers also make them attractive candidates for incorporation into targeted drug-delivery strategies. However, this potential application has not been evaluated in the present study and should therefore be considered a future perspective. Aptamers are well-documented for their high specificity and affinity toward their targets, with minimal associated side effects (41, 42). In 2004, the U.S. Food and Drug Administration (FDA) granted approval for the first aptamer-based therapeutic agent, pegaptanib sodium (Macugen^®^), for the treatment of age-related macular degeneration. More recently, in 2023, the FDA approved avacincaptad pegol (Izervay™), an RNA aptamer developed for the management of geographic atrophy secondary to age-related macular degeneration (43). Although these precedents support the therapeutic potential of aptamers as a molecular targeting platform, further studies are required to comprehensively evaluate the specificity, stability, safety, and potential off-target effects of the aptamers investigated in the present study. Collectively, the findings presented here provide a foundation for further characterization of the AptaBreast panel and its potential application as a precision-targeting platform for TNBC.

Within the field of oncology, AS1411 was the first aptamer to undergo clinical evaluation for cancer therapy. Subsequently, multiple other aptamers have entered clinical trials (44). However, to date, no aptamer-based therapeutics have obtained regulatory approval for oncological applications. This underscores the necessity for further research to elucidate the potential of aptamers in cancer therapy and to advance their clinical translation.

Aptamers represent a promising class of therapeutic agents with potential for targeted applications in cancer treatment. Given their ability to selectively recognize molecular targets, they may have potential for incorporation into future therapeutic strategies, including combination approaches with existing treatments. However, the therapeutic safety, efficacy, and potential clinical benefits of the aptamers investigated in the present study remain to be established. Further studies using more complex *in vitro* models and *in vivo* systems will be necessary to determine their therapeutic potential. In parallel, ongoing studies will focus on the identification of the molecular targets recognized by these aptamers and the elucidation of the mechanisms of action underlying the effects observed in the present study, providing further insight into their biological activity and translational potential applicability in TNBC. A schematic overview summarizing the overall findings and proposed model is presented in **Figure 6**. 

**Figure 6:**
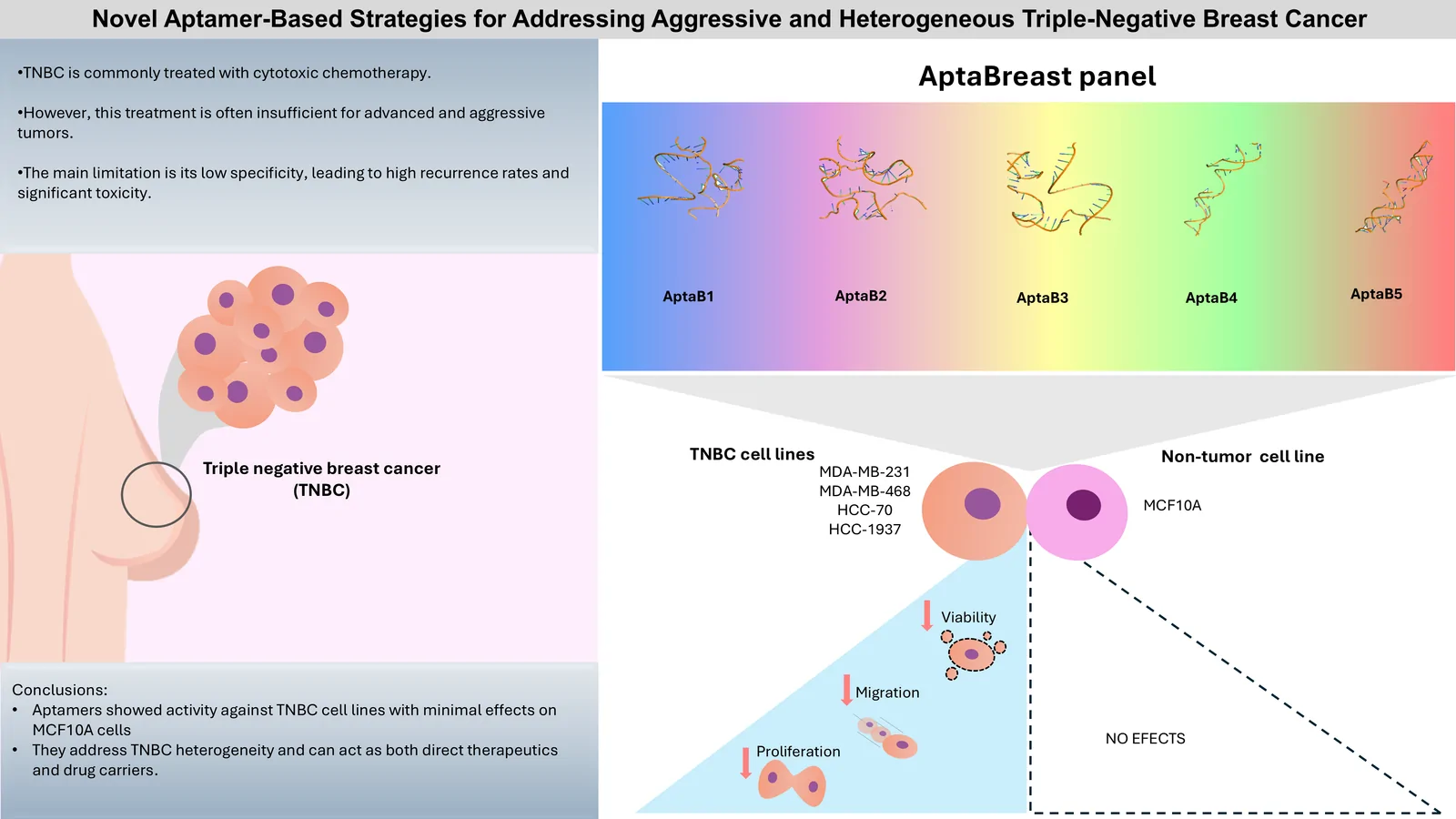
Summary schematic of the action of the panel of aptamers (AptaB1–AptaB5). MDA-MB-231 (tumor) and MCF10A (non-tumoral) breast epithelial cell lines were treated with the AptaBreast aptamer panel (AptaB1–AptaB5). The aptamers exhibited activity across TNBC cell lines, including antiproliferative and cell death effects in MDA-MB-231 cells, while showing limited effects on non-tumoral MCF10A cells.

## 5 Conclusion

This study demonstrates the biological activity of the AptaBreast panel (AptaB1–AptaB5) against TNBC cells, with effects on cell viability, proliferation, migration, and colony formation across multiple intrinsic subtypes. The absence of significant effects on MCF10A cells suggests limited effects on this non-tumoral cell model under the experimental conditions evaluated. However, further studies are required to validate these findings in more complex models and *in vivo* systems and to comprehensively assess their therapeutic efficacy and safety. In parallel, further investigation into the molecular targets recognized by these aptamers and the mechanisms underlying their biological effects will be essential to better define their therapeutic potential. Overall, these findings support further investigation of the AptaBreast panel, including the characterization of its molecular targets and mechanisms of action, as well as preclinical evaluation of its potential for TNBC-targeted strategies.

## 6 Limitations of the study

Although the AptaBreast panel demonstrated promising biological effects in TNBC cell lines, important steps remain to establish its translational potential. First, the molecular targets of the aptamers and the mechanisms underlying their biological effects remain to be identified and validated. In addition, the functional assays were predominantly performed using established TNBC cell lines, with migration and colony-formation assays evaluated only in the MDA-MB-231 model. Further studies using more complex and clinically relevant models, including three-dimensional and patient-derived tumor models, are therefore warranted. The aptamers will also require optimization to improve their stability and functional properties before advancing to preclinical studies. Finally, additional studies will be necessary to evaluate their specificity, biodistribution, toxicity, and efficacy *in vivo*. These studies will be essential to determine whether the biological effects observed *in vitro* can be translated into a viable therapeutic strategy for TNBC.

Data are contained within the article.

## 7 Patents

The aptamer sequences are protected under patent filing PCT/BR2022/050356.

## Data Availability

The raw data supporting the conclusions of this article will be made available by the authors, without undue reservation.

## References

[1] Wilkinson L Gathani T . Understanding breast cancer as a global health concern. Br J Radiol. (2022) 95:20211033. doi: 10.1259/bjr.20211033 34905391 PMC8822551

[2] Parker JS Mullins M Cheang MC Leung S Voduc D Vickery T . Supervised risk predictor of breast cancer based on intrinsic subtypes. J Clin Oncol. (2009) 27:1160–7. doi: 10.1200/JCO.2008.18.1370 19204204 PMC2667820

[3] Metzger-Filho O Tutt A de Azambuja E Saini KS Viale G Loi S . Dissecting the heterogeneity of triple-negative breast cancer. J Clin Oncol. (2012) 30:1879–87. doi: 10.1200/JCO.2011.38.2010 22454417

[4] Li Y Zhan Z Yin X Fu S Deng X . Targeted therapeutic strategies for triple-negative breast cancer. Front Oncol. (2021) 11:4517. doi: 10.3389/fonc.2021.731535 PMC8581040 34778045

[5] Lehmann BD Jovanović B Chen X Estrada MV Johnson KN Shyr Y . Refinement of triple-negative breast cancer molecular subtypes: implications for neoadjuvant chemotherapy selection. PloS One. (2016) 11:e0157368. doi: 10.1371/journal.pone.0157368 27310713 PMC4911051

[6] Wu Q Siddharth S Sharma D . Triple-negative breast cancer: a mountain yet to be scaled despite the triumphs. Cancers (Basel). (2021) 13:3697. doi: 10.3390/cancers13153697 34359598 PMC8345029

[7] He S Du Y Tao H Duan H . Advances in aptamer-mediated targeted delivery system for cancer treatment. Int J Biol Macromol. (2023) 238:124173. doi: 10.1016/j.ijbiomac.2023.124173 36965552

[8] Retecki K Seweryn M Graczyk-Jarzynka A Bajor M . The immune landscape of breast cancer: strategies for overcoming immunotherapy resistance. Cancers (Basel). (2021) 13:6012. doi: 10.3390/cancers13236012 34885122 PMC8657247

[9] Yang LF Ling M Kacherovsky N Pun SH . Aptamers 101: aptamer discovery and *in vitro* applications in biosensors and separations. Chem Sci. (2023) 14:4961–78. doi: 10.1039/d3sc00308a PMC10189874 37206388

[10] Wu YX Kwon YJ . Aptamers: the "evolution" of SELEX. Methods. (2016) 106:21–8. doi: 10.1016/j.ymeth.2016.06.015 27109056

[11] Zhuo Z Yu Y Wang M Li J Zhang Z Liu J . Recent advances in SELEX technology and aptamer applications in biomedicine. Int J Mol Sci. (2017) 18:2142. doi: 10.3390/ijms18102142 29036890 PMC5666824

[12] Zhang Y Lai BS Juhas M . Recent advances in aptamer discovery and applications. Molecules. (2019) 24:941. doi: 10.3390/molecules24050941 30866536 PMC6429292

[13] Macdonald J Denoyer D Henri J Jamieson A Burvenich IJG Pouliot N . Bifunctional aptamer-doxorubicin conjugate crosses the blood-brain barrier and selectively delivers its payload to EpCAM-positive tumor cells. Nucleic Acid Ther. (2020) 30:117–28. doi: 10.1089/nat.2019.0836 32027209 PMC7133447

[14] Mahmoudian F Ahmari A Shabani S Sadeghi B Fahimirad S Fattahi F . Aptamers as an approach to targeted cancer therapy. Cancer Cell Int. (2024) 24:108. doi: 10.1186/s12935-024-03237-7 38493153 PMC10943855

[15] de Araújo NS Moreira ADS Abreu RDS Junior VV Antunes D Mendonça JB . Aptamer-based recognition of breast tumor cells: a new era for breast cancer diagnosis. Int J Mol Sci. (2024) 25:840. doi: 10.3390/ijms25020840 38255914 PMC10815801

[16] Pinto-Díez C Ferreras-Martín R Carrión-Marchante R Klett-Mingo JI García-Hernández M Pérez-Morgado MI . An optimized MNK1b aptamer, apMNKQ2, and its potential use as a therapeutic agent in breast cancer. Mol Ther Nucleic Acids. (2022) 30:553–68. doi: 10.1016/j.omtn.2022.02.017 36457699 PMC9705393

[17] Garrido-Castro AC Lin NU Polyak K . Insights into molecular classifications of triple-negative breast cancer: improving patient selection for treatment. Cancer Discov. (2019) 9:176–98. doi: 10.1158/2159-8290.CD-18-1177 30679171 PMC6387871

[18] da Silva JL Cardoso Nunes NC Izetti P de Mesquita GG de Melo AC . Triple-negative breast cancer: a thorough review of biomarkers. Crit Rev Oncol Hematol. (2020) 145:102855. doi: 10.1016/j.critrevonc.2019.102855 31927455

[19] Wahba HA El-Hadaad HA . Current approaches in treatment of triple-negative breast cancer. Cancer Biol Med. (2015) 12:106–16. doi: 10.7497/j.issn.2095-3941.2015.0030 26175926 PMC4493381

[20] Nedeljković M Damjanović A . Mechanisms of chemotherapy resistance in triple-negative breast cancer—how we can rise to the challenge. Cells. (2019) 8:957. doi: 10.3390/cells8090957 31443516 PMC6770896

[21] Yin L Duan JJ Bian XW Yu SC . Triple-negative breast cancer molecular subtyping and treatment progress. Breast Cancer Res. (2020) 22:61. doi: 10.1186/s13058-020-01296-5 32517735 PMC7285581

[22] Liu CY Hung MH Wang DS Chu PY Su JC Teng TH . Tamoxifen induces apoptosis through cancerous inhibitor of protein phosphatase 2A-dependent phospho-Akt inactivation in estrogen receptor-negative human breast cancer cells. Breast Cancer Res. (2014) 16:431. doi: 10.1186/s13058-014-0431-3 25228280 PMC4303112

[23] Oltra SS Peña-Chilet M Martinez MT Tormo E Cejalvo JM Climent J . miRNA expression analysis: cell lines HCC1500 and HCC1937 as models for breast cancer in young women and miR-23a as a poor prognostic biomarker. Breast Cancer (Auckl). (2020) 14:1178223420977845. doi: 10.1177/1178223420977845 33311984 PMC7716059

[24] Mladkova J Sanda M Matouskova E Selicharova I . Phenotyping breast cancer cell lines EM-G3, HCC1937, MCF7 and MDA-MB-231 using 2-D electrophoresis and affinity chromatography for glutathione-binding proteins. BMC Cancer. (2010) 10:1–10. doi: 10.1186/1471-2407-10-1 20731849 PMC2933630

[25] Holliday DL Speirs V . Choosing the right cell line for breast cancer research. Breast Cancer Res. (2011) 13:215. doi: 10.1186/bcr2889 21884641 PMC3236329

[26] Lehmann BD Pietenpol JA . Identification and use of biomarkers in treatment strategies for triple-negative breast cancer subtypes. J Pathol. (2014) 232:142–50. doi: 10.1002/path.4280 24114677 PMC4090031

[27] Casacuberta-Serra S González-Larreategui Í Capitán-Leo D Soucek L . MYC and KRAS cooperation: from historical challenges to therapeutic opportunities in cancer. Signal Transduct Target Ther. (2024) 9:205. doi: 10.1038/s41392-024-01792-w 39164274 PMC11336233

[28] Lehmann BD Bauer JA Chen X Sanders ME Chakravarthy AB Shyr Y . Identification of human triple-negative breast cancer subtypes and preclinical models for selection of targeted therapies. J Clin Invest. (2011) 121:2750–67. doi: 10.1172/JCI45014 21633166 PMC3127435

[29] Wang DY Jiang Z Ben-David Y Woodgett JR Zacksenhaus E . Molecular stratification within triple-negative breast cancer subtypes. Sci Rep. (2019) 9:19107. doi: 10.1038/s41598-019-55620-w 31836816 PMC6911070

[30] Masuda H Baggerly KA Wang Y Zhang Y Gonzalez-Angulo AM Meric-Bernstam F . Differential response to neoadjuvant chemotherapy among seven triple-negative breast cancer molecular subtypes. Clin Cancer Res. (2013) 19:5533–40. doi: 10.1158/1078-0432.CCR-13-0799 23948975 PMC3813597

[31] Li X Yang J Peng L Sahin AA Huo L Ward KC . Triple-negative breast cancer has worse overall and cause-specific survival than non-triple-negative breast cancer. Breast Cancer Res Treat. (2017) 161:279–87. doi: 10.1007/s10549-016-4059-6 27888421

[32] Kim S Park JM Park S Jung E Ko D Park M . Suppression of TNBC metastasis by doxazosin, a novel dual inhibitor of c-MET/EGFR. J Exp Clin Cancer Res. (2023) 42:292. doi: 10.1186/s13046-023-02793-5 37924112 PMC10625208

[33] Gupta GP Massagué J . Cancer metastasis: building a framework. Cell. (2006) 127:679–95. doi: 10.1016/j.cell.2006.11.001 17110329

[34] Fares J Fares MY Khachfe HH Salhab HA Fares Y . Molecular principles of metastasis: a hallmark of cancer revisited. Signal Transduct Target Ther. (2020) 5:28. doi: 10.1038/s41392-020-0134-x 32296047 PMC7067809

[35] Franken NA Rodermond HM Stap J Haveman J van Bree C . Clonogenic assay of cells *in vitro*. Nat Protoc. (2006) 1:2315–9. doi: 10.1038/nprot.2006.339 17406473

[36] Rakha EA Reis-Filho JS Baehner F Dabbs DJ Decker T Eusebi V . Breast cancer prognostic classification in the molecular era: the role of histological grade. Breast Cancer Res. (2010) 12:207. doi: 10.1186/bcr2607 20804570 PMC2949637

[37] Leon-Ferre RA Goetz MP . Advances in systemic therapies for triple-negative breast cancer. BMJ. (2023) 381:e071674. doi: 10.1136/bmj-2022-071674 37253507

[38] Won KA Spruck C . Triple-negative breast cancer therapy: current and future perspectives (review). Int J Oncol. (2020) 57:1245–61. doi: 10.3892/ijo.2020.5156 33174058 PMC7646583

[39] Abramson VG Lehmann BD Ballinger TJ Pietenpol JA . Subtyping of triple-negative breast cancer: implications for therapy. Cancer. (2015) 121:8–16. doi: 10.1002/cncr.28914 25043972 PMC4270831

[40] Asleh K Riaz N Nielsen TO . Heterogeneity of triple-negative breast cancer: current advances in subtyping and treatment implications. J Exp Clin Cancer Res. (2022) 41:265. doi: 10.1186/s13046-022-02497-7 36050786 PMC9434975

[41] Sundaram P Kurniawan H Byrne ME Wower J . Therapeutic RNA aptamers in clinical trials. Eur J Pharm Sci. (2013) 48:259–71. doi: 10.1016/j.ejps.2012.10.006 23142634

[42] Kovacevic KD Gilbert JC Jilma B . Pharmacokinetics, pharmacodynamics and safety of aptamers. Adv Drug Delivery Rev. (2018) 134:36–50. doi: 10.1016/j.addr.2018.08.004 30321620

[43] Santarpia G Carnes E . Therapeutic applications of aptamers. Int J Mol Sci. (2024) 25:6742. doi: 10.3390/ijms25126742 38928448 PMC11204156

[44] Gao F Yin J Chen Y Guo C Hu H Su J . Recent advances in aptamer-based targeted drug delivery systems for cancer therapy. Front Bioeng Biotechnol. (2022) 10:972933. doi: 10.3389/fbioe.2022.972933 36051580 PMC9424825

